# American Society of Dental Surgeons

**Published:** 1848-07

**Authors:** 


### Miscellaneous Notices.

*American Society of Dental Surgeons.*—Before another number of the the Journal will have been issued, this Association will have held its Ninth Annual Meeting, and we trust the proceedings on the occasion will be both interesting and instructive to the members who may be present, and that it may be well attended. From the proceedings of the last meeting, we have reason to believe, that the next will have a more practical bearing than any of the preceding ones, and we feel assured that it will, if the Committee on Practical Dentistry discharge its duties in a manner we have a right to expect from the well known abilities of the gentlemen who compose it. The improvements constantly being made in Practical Dentistry, afford ample material for an elaborate and interesting report, and we trust that the members of the Society have aided the Committee, by furnishing it with such contributions as they have had it in their power to make. Without such co-operation on thier part, it will not be expected that the Committee will be able to make as full a report as they otherwise would.

But apart from the report of this Committee, the whole of the first day of the next meeting of the Society is to be devoted to an interchange of views, on one of the most important operations in Dental Surgery, namely: filling teeth, and in the discussion of the subject, the experience and opinions of the different members will doubtless be elicited, both with regard to the indications for, and the manner of, performing it. We hope, therefore, that the meeting may be well attended, and that all who go, may do so with the expectation, both of profiting and being profited. Every one who goes, should be prompted by either one or both of these motives—to be either teacher or pupil, or both. As he may freely receive whatever information may be communicated, let him also freely impart. And in seeking a high standard of practical excellence, let no one overlook the importance of a thorough theoretical knowledge of every thing connected, either remotely or immediately, with this department of medico-chirurgical science.

Finally, as the insignia of the Society is a polyandrian column, based upon a rock, and surmonted by the lamp of science, let the members, actuated by one common feeling, unitedly contribute, to the utmost of their abilities, to build it up so firmly upon the rock of scientific truth, that the lashings of the waves of envy and discontent, shall be able to make no impression upon it.

